# Evaluation of the Loop-Mediated Isothermal Amplification (LAMP) Technique in Swab Samples from Ulcerated Cutaneous Lesions Compared with Conventional Diagnostic Methods for American Tegumentary Leishmaniasis in Patients Treated at a Reference Center in Rio de Janeiro, Brazil

**DOI:** 10.3390/tropicalmed11070207

**Published:** 2026-07-22

**Authors:** Elizabeth Cristina Araújo Ferreira Faulhaber, Daniel Moreira de Avelar, Liliane de F. Antonio Oliveira, Luciana de F. Campos Miranda, Cintia Xavier de Mello, Gabrielle Baldan Stanciola, Luanna da S. Ventura, Marcelo Rosandiski Lyra, Caio Thomaz de Lima e Silva, Aline Fagundes da Silva, Rosely Maria Zancope-Oliveira, Maria Inês Fernandes Pimentel, Andreza Pain Marcelino

**Affiliations:** 1 Laboratório de Pesquisa e Vigilância em Leishmanioses, Instituto Nacional de Infectologia Evandro Chagas (INI), Fundação Oswaldo Cruz (Fiocruz), Rio de Janeiro 21040-900, Rio de Janeiro, Brazil; elizabethcafs@gmail.com (E.C.A.F.F.); stanciolagabrielle@gmail.com (G.B.S.); luannaventwra@gmail.com (L.d.S.V.); marcelolyradermato@hotmail.com (M.R.L.); caiotls@yahoo.com.br (C.T.d.L.e.S.); aline.fagundes@ini.fiocruz.br (A.F.d.S.); maria.pimentel@ini.fiocruz.br (M.I.F.P.); 2 Grupo de Estudos em Leishmanioses, Instituto René Rachou, Fundação Oswaldo Cruz (Fiocruz), Belo Horizonte 30190-002, Minas Gerais, Brazil; daniel.avelar@fiocruz.br; 3 Laboratório de Epidemiologia Clínica, Instituto Nacional de Infectologia Evandro Chagas (INI), Fundação Oswaldo Cruz (Fiocruz), Rio de Janeiro 21040-900, Rio de Janeiro, Brazil; liliane.fatima@ini.fiocruz.br; 4 Laboratório de Referência em Leishmanioses, Instituto Carlos Chagas, Fundação Oswaldo Cruz (Fiocruz), Curitiba 81350-010, Paraná, Brazil; luciana.campos@fiocruz.br; 5 Laboratório Interdisciplinar de Pesquisas Médicas, Instituto Oswaldo Cruz (IOC), Fundação Oswaldo Cruz (Fiocruz), Rio de Janeiro 21040-900, Rio de Janeiro, Brazil; cintia.mello@ioc.fiocruz.br; 6 Laboratório de Micologia, Instituto Nacional de Infectologia Evandro Chagas (INI), Fundação Oswaldo Cruz (Fiocruz), Rio de Janeiro 21040-900, Rio de Janeiro, Brazil; rosely.zancope@ini.fiocruz.br

**Keywords:** cutaneous leishmaniasis, molecular methods, diagnosis, swab

## Abstract

Cutaneous leishmaniasis (CL) is a vector-borne disease caused by *Leishmania* spp. Molecular methods such as conventional PCR (cPCR) and real-time PCR (qPCR) show high accuracy but remain restricted to reference centers. Loop-mediated isothermal amplification (LAMP) enables rapid DNA amplification under isothermal conditions. LAMP targeting 18S rDNA using WarmStart^®^ Colorimetric LAMP 2× Master Mix and Bst 2.0 DNA Polymerase with SYBR^®^ Green I (BstSg) was evaluated in swab samples (extracted by kit—SW-kit and direct boil—SW-DB) and specimens collected through biopsy from ulcerated lesions of patients attending a reference center in Rio de Janeiro, Brazil. The reference standard was defined as at least one positive parasitological or molecular (cPCR) test plus clinical confirmation. Analytical sensitivity reached 10 fg with WS and 10 pg with BstSg. The diagnostic accuracy of LAMP ranged from 51.8% to 78.6%, with sensitivity from 52.5% to 81% and specificity from 39.6% to 89.4%. LAMP, particularly when applied to swab samples with DNA extracted using the SW-kit protocol and amplified with BstSg, showed promising diagnostic accuracy and operational feasibility as a less invasive approach for CL diagnosis, eliminating the need for skin biopsy and reducing patient discomfort during sample collection.

## 1. Introduction

Cutaneous leishmaniasis (CL) is a neglected tropical disease (NTD) caused by protozoan parasites of the genus *Leishmania* transmitted by phlebotomine sandflies. It is endemic in 99 countries across tropical, subtropical, and Mediterranean regions, with an estimated 600,000–1 million new cases annually [1]. In the Americas, it is widely distributed from the southern United States to the northern Argentina, excluding the Caribbean islands and Chile, with 85% of CL cases concentrated in Brazil, Colombia, and Peru [1].

The effectiveness of treatment of CL is dependent upon accurate and timely diagnosis, which is essential for cure and the prevention of disfiguring sequelae [2,3]. Current diagnostic approaches include serological, histopathological, parasitological (culture in appropriate media and direct examination), and molecular methods such as conventional PCR (cPCR) and real-time PCR (qPCR). Although molecular assays provide high accuracy, they require specialized laboratory infrastructure, limiting their availability to reference centers [4,5].

The World Health Organization highlights CL as one of the 20 priority NTDs due to its substantial impact on health, social well-being, and economic systems. Considering these challenges and the need for simplified diagnostic tools, loop-mediated isothermal amplification (LAMP) has emerged as a promising alternative, enabling rapid DNA amplification under isothermal conditions. In this study, we evaluated the performance of the LAMP technique using 18S rDNA primers [6] indifferent clinical samples and DNA extraction/amplification protocols for the diagnosis of CL.

## 2. Materials and Methods

### 2.1. Study Participants and Reference Standard

This study evaluated the diagnostic performance of the LAMP assay for CL. Clinical samples were collected from patients attending the outpatient clinic of the Instituto Nacional de Infectologia Evandro Chagas, Fundação Oswaldo Cruz (INI/Fiocruz), Rio de Janeiro, Brazil, between October 2021 and December 2024.

Participants were classified into two groups. Group I (cases) included patients with a confirmed diagnosis of CL, established by at least one positive parasitological test (culture in appropriate media, direct microscopy, histopathology, or immunohistochemistry with parasite detection) or at least one positive molecular test (conventional PCR), combined with clinical confirmation and exclusion of differential diagnoses. Group II (controls) comprised patients with initially suspected CL but with a different final diagnosis.

Inclusion criteria were patients aged ≥13 years presenting with ulcerated skin lesions (regardless of number), with clinical suspicion of CL, who attended the INI/Fiocruz outpatient clinic during the study period and agreed to participate in the study by signing the Informed Consent Form. Exclusion criteria included prior treatment for CL and negative samples for endogenous beta-globin gene control, indicating inadequate DNA quality or extraction failure, which could compromise the performance of the molecular assays.

The minimum required sample size to estimate diagnostic accuracy parameters was calculated using the formula proposed by Banoo et al. [7] (n ≥ z^2^ × p [1 − p]/x^2^), where n is the number of positive or negative samples, z = 1.96, p represents sensitivity or specificity, and x is the confidence interval (CI). Assuming a 90% CI and previously reported sensitivity (86%) and specificity (92.6%) for 18S LAMP [8], a minimum of 46 positive and 26 negative samples were required.

### 2.2. Sample Collection and Storage

All clinical samples were obtained under aseptic conditions. Prior to collection, the lesion area was cleaned with 70% alcohol and 2% chlorhexidine. Swab samples: specimens were first collected using sterile cotton swabs. Each swab was then placed in a sterile 2 mL microtube without preservatives and stored at −20 °C until DNA extraction. Direct examination samples: the same lesion was subsequently scraped at the inner face of the edge using a size 15 scalpel. Two smears were prepared on glass slides for direct microscopic examination, stored at room temperature until fixation and staining. Biopsy samples: following local anesthesia with 2% lidocaine, a 5–6 mm circular scalpel (punch) biopsy was obtained from the same lesion. From this fragment, two imprint smears were prepared on glass slides for microscopic examination and stored at room temperature until fixation and staining. The remaining biopsy material was divided into three portions: one placed in a sterile, dry 0.5 mL microtube and stored at −20 °C for DNA extraction; one placed in a sterile 2 mL microtube containing buffered saline supplemented with 1200 U of penicillin, 100 μg of streptomycin, and 100 μg of 5′-fluorocytosine, stored at 2–8 °C for 24 h before culture in appropriate media; one preserved in 10% formalin at room temperature for up to 24 h for histopathological/immunohistochemistry examination.

### 2.3. Reference Tests

Given the lack of a single definitive gold standard for cutaneous leishmaniasis, a combination of parasitological, molecular, and clinical criteria was employed to reduce the risk of misclassification among cases and controls.

#### 2.3.1. Culture

For indirect parasitological diagnosis, tissue fragments obtained thorough skin biopsy were processed and inoculated into a biphasic medium composed of McNeal, Novy, and Nicolle (NNN, solid phase) and Schneider’s *Drosophila* (Sigma Aldrich, St. Louis, MO, USA), media supplemented with 3.0 mL penicillin (stock solution with 63,000 Units/mL/Sigma Aldrich, St. Louis, MO, USA) and 4.0 mL Streptomycin (stock solution with 50,000 µg/mL/Sigma Aldrich, St. Louis, MO, USA) for each liter of culture medium and add 10% inactivated and sterile fetal bovine serum (FBS-Sigma Aldrich-Paraguay). Cultures were incubated at 26–28 °C and examined under a light microscope for the presence of living mobile promastigotes, following the protocol available at https://dx.doi.org/10.17504/protocols.io.n92ldry5og5b/v1 [9]. Isolates were further characterized by multilocus enzyme electrophoresis (MLEE) using a protocol adapted from Cupolillo et al. and standardized by the Laboratory of Clinical Research and Surveillance in Leishmaniasis, INI/Fiocruz [10].

#### 2.3.2. Direct Examination

Direct parasitological diagnosis was performed according to the Laboratory of Clinical Research and Surveillance in Leishmaniasis protocol [11]. Imprint smears were prepared by pressing the biopsy fragment onto glass slides, fixed with methanol, and stained with May–Grünwald–Giemsa for 15 min. Amastigotes were visualized through light microscopy with 1000× magnification and oil immersion. For scrape smears, material from the inner face of the edge of the lesion was scraped with a scalpel, placed on a slide, fixed with methanol and stained with Giemsa.

#### 2.3.3. Histopathology

Histopathological examination was performed following the protocols of the Pathological Anatomy Service of INI/Fiocruz [12]. Biopsy fragments were fixed in 10% formalin, embedded in paraffin, sectioned, and stained with hematoxylin and eosin (H&E). Slides were examined though light microscopy for detection of amastigotes.

#### 2.3.4. Immunohistochemistry

Immunohistochemistry was performed following the protocols of the Pathological Anatomy Service of INI/Fiocruz [12]. Anti-*Leishmania* serum was produced at the Laboratory of Clinical Research and Surveillance in Leishmaniasis by immunization of albino rabbits inoculated with four doses of soluble protein extracts of promastigote forms of *Leishmania* (*Leishmania*) *infantum* (MHOM/BR/1974/PP75). Tissue fragments were submitted to inhibition of endogenous peroxidase with H_2_O_2_ and methanol, antigen retrieval with citrate buffer, at pH 9.0 in a pressure cooker for three min after boiling, followed by cooling, and inhibition of nonspecific binding with Molico^®^ milk powder and bovine serum albumin. The specimens were incubated overnight in a refrigerator at 4 °C with the primary antibody (anti-*Leishmania* hyperimmune serum) diluted 1:800. The reaction was developed using the Universal DAKO LSAB + Detection System (catalog No. K0690, DAKO Corporation, Carpinteria, CA, USA) and using diaminobenzidine as chromogen.

#### 2.3.5. Conventional PCR (cPCR)

Molecular diagnosis was standardized according to Fagundes et al. [13], targeting a conserved region of the *Leishmania* kinetoplast DNA (kDNA) minicircle (120 bp).

DNA was extracted from biopsy tissue fragments using a silica column, the PureLink Genomic DNA Mini Kit (Invitrogen—Thermo Fisher Scientific, Waltham, MA, USA). A portion of the DNA was reserved for LAMP reactions, and another portion was reserved for cPCR. The cPCR reaction was performed using primers HM1 (5′-CCG CCC CTA TTT TAC ACC ACC CCC-3′), HM2 (5′-GGG GAG GGG CGT TCT GCG AA-3′), and HM3 (5′-GGC CCA CTA TAT TAC ACC AAC CCC-3′). PCR cycling conditions were initial denaturation at 95 °C for 15 min, followed by 30 cycles of 95 °C for 30 s, 57 °C for 30 s, and 72 °C for 10 min. Positive controls (human clinical samples) and negative controls (nuclease-free water) were included in each run.

### 2.4. LAMP

#### 2.4.1. Preparation of Reference DNA

Genomic DNA obtained from reference strains of *Leishmania* (*Viannia*) *braziliensis* (MHOM/BR/1975/M2903) (Lb), *L.* (*Leishmania*) *amazonensis* (IFLA/BR/19767/PH8) (La), and *L.* (*Leishmania*) *infantum* (MHOM/BR/1974/PP75) (Li), using the PureLink Genomic DNA Mini Kit served as positive controls. The analytical sensitivity of each LAMP protocol was determined using serial ten-fold dilutions (1 ng to 1 fg) of Lb genomic DNA. The objective of this analysis was to establish the limit of detection (LOD), defined as the lowest DNA concentration consistently detected in duplicate reactions. Since the LAMP assay generates qualitative binary results (positive/negative) based on colorimetric visualization and gel electrophoresis, quantitative calibration curves such as those commonly used in real-time PCR assays were not applicable. Analytical specificity was assessed using DNA samples (5 ng/µL) from *Paracoccidioides brasiliensis* (Pb), *Sporothrix schenckii* (Ss), *Sporothrix brasiliensis* (Sb), *Sporothrix globosa* (Sg), Lb), *L.* (*Viannia*) *lainsoni* (Ll), Li, *L.* (*Viannia*) *guyanensis* (Lg), La, and *L.* (*Viannia*) *shawi* (Ls).

#### 2.4.2. Standardization

Preliminary assays were performed to evaluate a set of 18S rDNA primers—F3 (GGG TGT TCT CCA CTC CAG A), B3 (CCA TGG CAG TCC ACT ACA C), FIP (TAC TGC CAG TGA AGG CAT TGG TGG CAA CCA TCG TGA C), and BIP (TGC GAA AGC CGG CTT GTT CCC ATC ACC AGC TGA TAG GGC), as described by Nzelu et al. [6]. The LAMP assay 1 (WS) was conducted in 25 µL of a reaction mixture consisting of a 1.6 µM concentration of each inner primer (FIP and BIP), 0.2 µM of each outer primer (F3 and B3), 12.5 µL of the WarmStart^®^ Colorimetric LAMP 2× Master Mix (New England Biolabs, Ipswich, MA, USA), 0.8 M betaine and 2 µL (200 pg) of Lb DNA (MHOM/BR/1975/M2903). The WS contains the colorimetric reagent phenol red, which changes color with proton release and a decrease in pH, turning yellow when amplification occurs (positive) and remaining pink in the absence of amplification (negative). The LAMP assay 2 (BstSg) was performed using the same primer concentrations as in assay 1, supplemented with 8 U of Bst 2.0 DNA Polymerase (New England Biolabs, USA), 1 mM DNPp, 0.8 M betaine, 20 mM Tris-HCl (pH 8.8), 10 mM KCl, 10 mM (NH_4_)_2_SO_4_, 8 mM MgSO_4_, 1% Tween 20, and 2 µL (200 pg) of Lb DNA. On the inner side of the tube cap, 1 μL of SYBR Green I 10,000×/DMSO (Invitrogen^®^), diluted 1:10, was added and incorporated into the reaction after amplification though manual mixing with the tube closed. This dye also allows naked-eye visualization, binding to amplified DNA when the reaction is positive (yellow) and showing an orange color in the absence of amplification (negative).

The reaction mixtures (WS and BstSg) were incubated for 30–60 min in a water bath (Novatécnica, model NT 248, Piracicaba, São Paulo, Brazil), with observations recorded at 10 min intervals. Results were visually assessed by two independent observers immediately after amplification. Observer 1 was a biologist trained in LAMP methodology at the René Rachou Institute, Fiocruz, and experienced in molecular diagnostics. Observer 2 was a biomedical sciences student with prior experience in molecular diagnostics, trained under the supervision of Observer 1. Each observer performed the evaluation independently, and results were subsequently compared. In cases of disagreement, a third observer, a veterinarian with expertise in diagnostic test validation and trained at the René Rachou Institute, Fiocruz, reviewed the archived image of the reaction to determine the result.

#### 2.4.3. Clinical Sample Testing

DNA extraction from clinical samples from one of the swabs (SW-kit) was performed using a silica column, the PureLink^®^ Genomic DNA Mini Kit (Thermo Fisher Scientific—Waltham, MA, USA), according to the manufacturer’s guidelines. In the other swab sample (SW-DB), extraction with the Direct Boil method was used [14], known as boiling. After extraction, DNA was stored at −20 °C until use. DNA samples were tested at different DNA concentrations to determine the optimal input. The extracted DNA was measured, and the purity ratio of 260/280 was established by spectrophotometry on the NanoDrop 2000 equipment (Thermo Fisher Scientific—Waltham, MA, USA). Positive controls (PC) consisted of DNA extracted from the Lb reference strain, negative controls (NC) included a clinical sample from a patient confirmed negative for leishmaniasis (tested in duplicate). A no-template control (NTC) with water was also included. To monitor both DNA integrity and the absence of PCR inhibitors, samples were tested for the human β-globin gene, used exclusively as an internal control for DNA extraction quality [15]. PCR amplification of the human β-globin gene was performed using primers PC03 (5′-ACACAACTGTGTTCACTAGC-3′) and PC04 (5′-CAACTTCATCCACGTTCACC-3′), generating a 110 bp amplicon. PCR was carried out with an initial denaturation at 94 °C for 5 min, followed by 30 cycles of 94 °C for 1 min, 55 °C for 2 min, and 72 °C for 1 min, with a final extension at 72 °C for 7 min. All assays were performed in duplicate.

### 2.5. Leishmania Species in Clinical Samples

The *Leishmania* species were characterized by multilocus enzyme electrophoresis (MLEE) using the enzyme systems glucose-6-phosphate dehydrogenase (G6PDH), phosphoglucose isomerase (GPI), nucleoside hydrolase (NH), phosphoglucomutase (PGM), and 6-phosphogluconate dehydrogenase (6PGDH), following a standardized protocol [10]. Parasite masses obtained from positive cultures were used for analysis. The electrophoretic profiles of *Leishmania* isolates from clinical samples were compared with those of reference strains of *L. braziliensis* (MHOM/BR/1975/M2903) and *L. infantum* (MHOM/BR/1974/PP75).

### 2.6. Statistical Analysis

Statistical analysis was performed in two steps. In the first step, clinical, laboratory, and demographic characteristics were described using frequencies for categorical variables (sex, location of the lesion, comorbidities, laboratory results, clinical form, treatment response) and measures of central tendency and dispersion for continuous variables (age, number of lesions, disease duration). For the analysis comparing clinical and demographic data between cases and controls, the Pearson’s chi-square test was used for categorical variables, and the Wilcoxon test was used for continuous variables. The normality of continuous variables was assessed using the Shapiro test. In the second, the accuracy of the evaluated test was assessed against the reference standard by calculating sensitivity, specificity, and overall accuracy. Reliability was evaluated using simple agreement. Analyses were conducted in R (version 4.4.2), with a 95% confidence interval and a significant level of 5% (*p* < 0.05).

### 2.7. Ethical Aspects

The procedures for this study were approved by the Research Ethics Committee (CEP) of INI/Fiocruz (CAAE: 51686621.9.0000.5262.211) and were performed in accordance with the ethical standards of human clinical research.

## 3. Results

### 3.1. Study Participants

**Figure 1:**
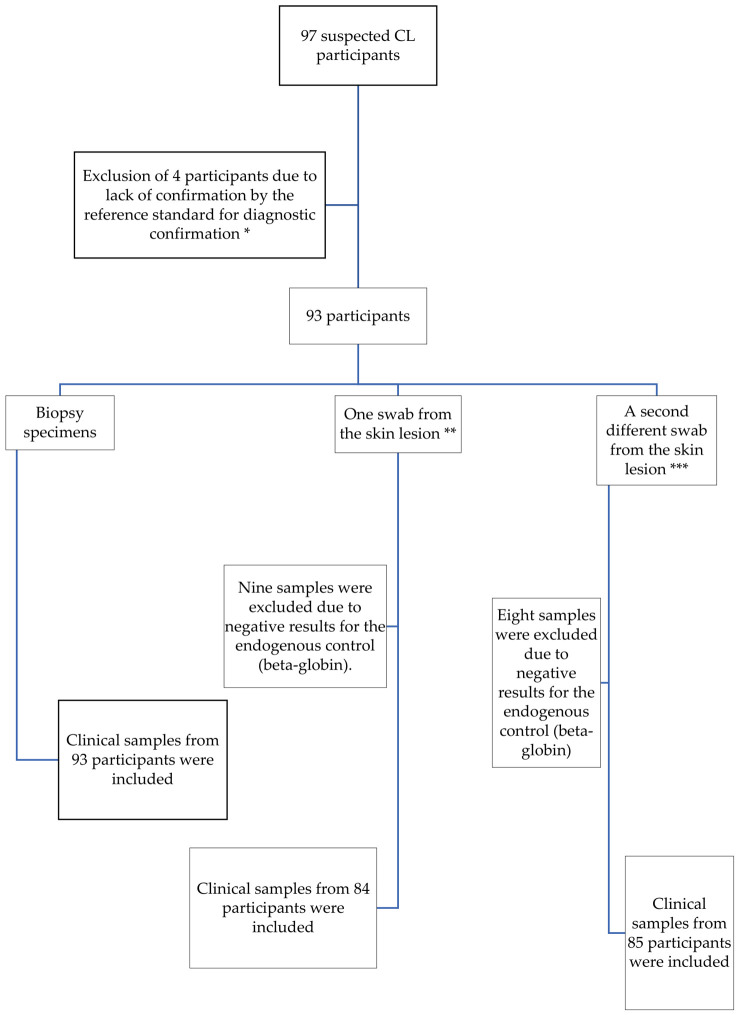
Inclusion of clinical samples in the study according to predefined methodological criteria. * At least one positive parasitological test (culture, direct examination, histopathology, or immunohistochemistry) or one positive molecular test (conventional PCR) with clinical confirmation, alongside negative results for differential diagnoses. ** DNA was extracted using the PureLink^®^ Genomic DNA Mini Kit. *** DNA was extracted using the boiling method.

Ninety-seven patients suspected of CL were evaluated at the outpatient clinic of the Leishmaniasis Clinical Research and Surveillance Laboratory. Some participants were excluded for not meeting pre-defined criteria based on reference and diagnostic quality test results (endogenous beta-globin gene). Figure 1 shows the inclusion process of participants and clinical samples in this study.

Of the 93 participants included in the study, 42 were classified as cases. Among the patients in the control group (those with a final diagnosis other than leishmaniasis), we observed cases of sporotrichosis (23/51), pyoderma (8/51), paracoccidioidomycosis (3/51), scabies (1/51), histoplasmosis (1/51), mycobacteriosis (1/51), and other non-infectious conditions (14/51). Table 1 presents the main clinical and demographic characteristics of the participants included in the study.

### 3.2. Standardization of LAMP Assay

The optimal incubation time was 40 min for WS and 60 min for BstSg. The optimal incubation temperature for all protocols was 64 °C (±1 °C). The LOD of WS was 100 fg and that of BstSg was 10 pg, with consistent results observed in both duplicates. All negative clinical controls and no-template controls remained negative throughout the standardization and clinical evaluation phases.

**Figure 2:**
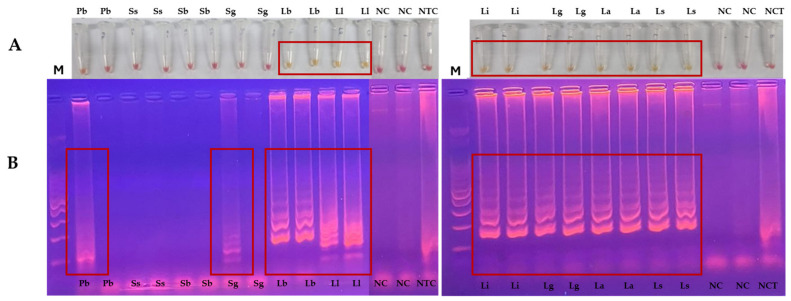
Specificity testing for the 18S rDNA target using the WarmStart^®^ Colorimetric LAMP 2× Master Mix kit and reference samples of *Paracoccidioides* spp., *Sporothrix* spp., and *Leishmania* spp. (**A**) Representative colorimetric LAMP reactions performed with DNA from *Paracoccidioides brasiliensis* (Pb), *Sporothrix schenckii* (Ss), *Sporothrix brasiliensis* (Sb), *Sporothrix globosa* (Sg), *Leishmania* (*Viannia*) *braziliensis* (Lb), *Leishmania* (*Viannia*) *lainsoni* (Ll), *Leishmania* (*Leishmania*) infantum (Li) *Leishmania* (*Viannia*) *guyanensis* (Lg), *Leishmania* (*Leishmania*) *amazonensis* (La), *Leishmania* (*Viannia*) *shawi* (Ls). NC, non-leishmaniasis control sample; NTC, no-template control. (**B**) Agarose gel electrophoresis of the corresponding amplification products. M, molecular weight marker.

**Figure 3:**
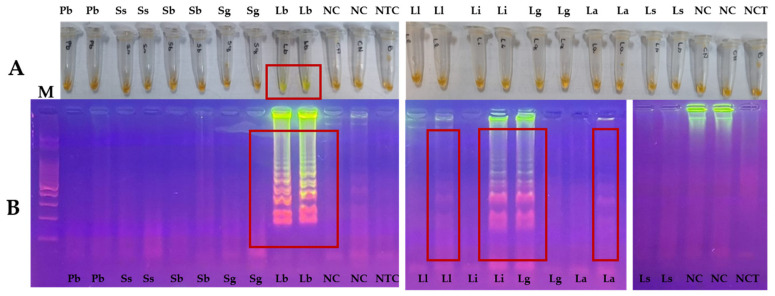
Specificity testing for the 18S rDNA target using Bst 2.0 DNA Polymerase and SYBR^®^ Green I dye, along with reference samples of *Paracoccidioides* sp., *Sporothrix* spp., and *Leishmania* sp. (**A**) Representative colorimetric LAMP reactions performed with DNA from *Paracoccidioides brasiliensis* (Pb), *Sporothrix schenckii* (Ss), *Sporothrix brasiliensis* (Sb), *Sporothrix globosa* (Sg), *Leishmania* (*Viannia*) *braziliensis* (Lb), *Leishmania* (*Viannia*) *lainsoni* (Ll), *Leishmania* (*Leishmania) infantum* (Li), *Leishmania* (*Viannia*) *guyanensis* (Lg), *Leishmania* (*Leishmania*) *amazonensis* (La) *Leishmania* (*Viannia*) *shawi* (Ls). NC, non-leishmaniasis control sample; NTC, no-template control. (**B**) Agarose gel electrophoresis of the corresponding amplification products. M, molecular weight marker.

The analytical specificity of the 18S rDNA LAMP assays was evaluated using DNA from different *Leishmania* species and other microorganisms potentially involved in the differential clinical diagnosis of cutaneous lesions. The 18S rDNA target combined with WS showed amplification for all *Leishmania* species tested, visible by visual inspection. Reactivity was also observed with Pb (visual inspection) and Sg (agarose gel electrophoresis only) in one duplicate reaction of each sample (Figure 2). During the specificity test performed with BstSg, a positive reaction for Lb was detected by visual inspection. In addition, agarose gel analysis showed amplification products for Li, Lg and one duplicate of La (Figure 3). The complete analytical specificity results, including visual inspection and agarose gel electrophoresis findings for all microorganisms tested, are presented in Supplementary Table S1.

In experiments evaluating different DNA dilutions of clinical samples, using BstSg with DNA extracted by the PureLink^®^ Genomic DNA Mini Kit (30 ng/µL; A260/280 = 1.81), amplification was observed in undiluted samples as well as in 1:10 and 1:20 dilutions. In contrast, for DNA extracted through boiling (223.9 ng/µL; A260/280 = 0.81), amplification was detected in the undiluted sample and in only one of the duplicates at 1:50 dilution. These findings indicate that the optimal condition for boiling-extracted DNA is its use in undiluted form. Performance analysis using the WS mix demonstrated that optimal results with both DNA extracted with the PureLink^®^ Genomic DNA Mini Kit and DNA obtained from swabs through boiling were achieved using undiluted samples. Based on these initial standardization findings, all subsequent LAMP reactions were performed using DNA samples without prior dilution.

### 3.3. Diagnostic Test Results

The median, interquartile range and minimum/maximum values for DNA quantification (ng/µL), as measured by microvolume spectrophotometer, as well as the 260/280 purity ratio for each clinical sample, are presented in Table 2. No significant differences in DNA quantification were observed between cases and controls across all sample types, including biopsy specimens (*p* = 0.1343; 0.1636), swab samples extracted using the PureLink^®^ Genomic DNA Mini Kit (SW-kit) (*p* = 0.2765; 0.6158), and swab samples extracted through the boiling method (SW-DB) (*p* = 0.133; 0.1104). However, a statistically significant difference (*p* < 0.001) was observed in DNA quantification between SW-DB and SW-kit when considering samples from all participants.

Sensitivity and specificity data for the 18S rDNA LAMP assays are presented in Table 3. Overall accuracy also differed among methods, ranging from 51.8% to 78.6% (95% CI) for 18S rDNA LAMP.

**Figure 4:**
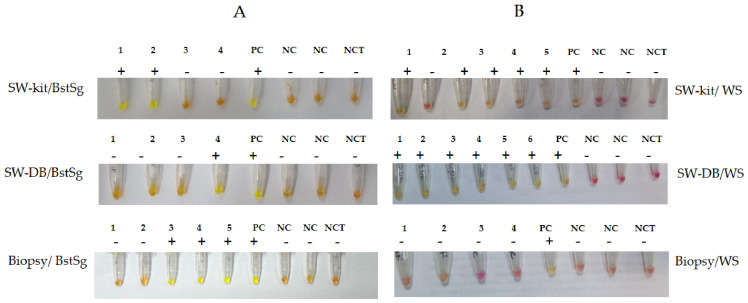
18S rDNA LAMP assay using Bst 2.0 DNA Polymerase and SYBR^®^ Green dye (**A**) and WarmStart^®^ Colorimetric LAMP 2× Master Mix kit (**B**), performed in duplicate on cutaneous lesion swab samples with DNA extracted using the PureLink^®^ Genomic DNA Mini Kit and Direct Boil and Biopsy from patients suspected of American tegumentary leishmaniasis, Rio de Janeiro. -kit: skin lesion swab with DNA extracted using the PureLink^®^ Genomic DNA Mini Kit; BstSg: tubes containing LAMP reactions with Bst 2.0 DNA Polymerase and SYBR^®^ Green I dye; SW-DB: skin lesion swab with DNA extracted by the direct boiling method (Direct Boil); WS: WarmStart^®^ Colorimetric LAMP 2× Master Mix. PC, positive control; NC, non-leishmaniasis control sample; NTC, no-template control; (1–6) clinical samples; (+) positive result; (-) negative result.

Figure 4 illustrates representative reactions of SW-kit and SW-DB in clinical samples.

Agreement between duplicates for the 18S rDNA LAMP assays using the WS mix was 91.7% for SW-kit samples and 90.6% for SW-DB samples. For the BstSg-based LAMP assays, agreement between duplicates was 79.8% for SW-kit and 80.0% for SW-DB samples. No discrepancies in the final classification of samples were observed between operators, indicating excellent inter-observer agreement.

Among the 42 participants with confirmed CL included in the study, 25 had positive culture results. Of these, 22 yielded sufficient parasite mass for isoenzyme analysis, which identification of the infecting species as *Leishmania* (*Viannia*) *braziliensis*.

## 4. Discussion

Accurate and opportune diagnosis of CL is essential to ensure appropriate treatment and prevent long-term sequelae [16]. The clinical presentation of CL is heterogeneous and may resemble other dermatological conditions, making laboratory exams crucial complements to clinical and epidemiological criteria. Available diagnostic tools can be broadly categorized into parasitological, serological, and molecular methods, each with inherent advantages and limitations [17].

Molecular techniques, particularly polymerase chain reaction (PCR), are recognized for their high sensitivity and specificity [18,19]. However, their implementation requires well-equipped laboratories, costly instrumentation, and trained personnel, which restricts their use in endemic, resource-limited settings. There is still a lack of standardized protocols and consensus regarding optimal molecular targets across laboratories [20]. In this context LAMP, first described by Notomi et al. [21], emerges as a promising alternative due to its operational simplicity, rapid turnaround, and the absence of a need for thermocyclers.

The study population showed a balanced distribution between cases and controls, as well as comparable demographic characteristics, minimizing potential selection bias and strengthening internal validity. A slight predominance of males among cases was observed, consistent with the known epidemiological profile of the disease in Brazil. Although the median duration of the lesions was similar between groups (approximately three months), longer disease duration was observed among controls, possibly reflecting delayed diagnosis or the presence of chronic dermatoses in this group.

Parasites isolated in culture that yielded sufficient biomass for analysis were identified as *Leishmania braziliensis* by MLEE, corroborating previous reports that identify *L. braziliensis* as the predominant species causing cutaneous leishmaniasis in the state of Rio de Janeiro [9].

The analytical sensitivity of LAMP observed in this study was 100 fg for WS and 10 pg for BstSg, consistent with previous reports using 18S rDNA and other targets such as HSP70 and kDNA, which have demonstrated good performance in samples with low parasite burden [13,17,22,23,24,25]. The 18S rDNA gene is widely used due to its high conservation among *Leishmania* species; however, this feature may also increase the risk of cross-reactivity with other trypanosomatids [19]. In our study, amplification was observed for all *Leishmania* species tested, as expected. Cross-reactivity was detected with *Paracoccidioides brasiliensis* and *Sporothrix globosa* in agarose gel electrophoresis in one duplicate when using WS, suggesting possible nonspecific amplification. Notably, these reactions were not detectable by visual inspection, indicating limited practical impact under field conditions. Nonetheless, these findings highlight potential specificity limitations associated with the 18S rDNA target, which may depend on primer design and reaction conditions.

Regarding sample processing, LAMP performance varied according to the DNA extraction method. DNA extracted using a commercial kit demonstrated consistent amplification across a range of dilutions, whereas boiling-extracted DNA yielded reliable results only when used undiluted. Based on these findings, all subsequent assays were performed using undiluted DNA to minimize the risk of false-negative results due to suboptimal DNA concentrations.

The use of lesion swabs represents a less invasive and more patient-friendly sampling approach, facilitating sample collection in remote settings and expanding access to molecular diagnosis without the need for biopsy procedures [26]. Although swab-based molecular diagnosis is well established, conventional PCR and qPCR still require complex infrastructure. In contrast, LAMP, offers significant potential for decentralization and implementation in primary healthcare settings, particularly when combined with simple heat sources and visual readouts [7].

In this study, the boiling extraction method yielded significantly higher DNA concentrations compared to the commercial kit, likely due to contaminants such as proteins and detergents (26/280: 0.65), that may artificially inflate spectrophotometric readings. Additionally, high temperatures may contribute to DNA degradation. Suboptimal DNA purity (outside the 1.6–2.0 range) may negatively impact diagnostic performance, particularly sensitivity [27]. Despite this, LAMP performance using boiling-extracted swab samples remains relevant given its easiness, low cost, and feasibility in resource-limited settings.

Overall, LAMP demonstrated higher sensitivity than direct microscopic examination (imprint), a diagnostic method widely used in endemic settings because of its simplicity, low cost, and rapid turnaround time [4,28]. While culture remains a good method for diagnosis due to its high specificity, its use is often restricted to reference laboratories because of its technical complexity.

The diagnostic performance of LAMP was strongly influenced by both sample type and reaction conditions. Previous studies have reported high analytical sensitivity for 18S rDNA LAMP assays, although data regarding their clinical performance remain relatively limited [27,29]. In comparison with published studies, the sensitivity observed for biopsy samples (60–81%) was lower than that reported in some investigations evaluating optimized LAMP assays, but remained consistent with the variability described in a recent systematic review, which highlighted substantial heterogeneity among studies due to differences in molecular targets, clinical specimens, extraction methods, and study design [27,29].

LAMP assays targeting different genomic regions, including 18S rDNA, HSP70 [25], and kDNA [22], have shown variable diagnostic performance depending on the molecular target, clinical sample type, and DNA extraction methodology. Assays targeting kDNA generally achieve high analytical sensitivity due to the large copy number of minicircle sequences, although concerns regarding species variability and specificity have been reported [30]. In contrast, HSP70-based assays have shown good diagnostic accuracy and improved species discrimination, but may present lower analytical sensitivity than multicopy targets [25]. The 18S rDNA target used in the present study represents a commitment between sensitivity and broad applicability, combining a high copy number with conservation across *Leishmania* species. These characteristics make it attractive for screening purposes, although they may also increase susceptibility to non-specific amplification under suboptimal reaction conditions.

Considerable heterogeneity exists among published LAMP studies, reflecting differences in study design, patient selection, clinical specimens, DNA extraction procedures, amplification protocols, and target genes. Consequently, direct comparisons between studies should be interpreted with caution. Nevertheless, our findings provide additional evidence regarding the performance of 18S rDNA-based LAMP assays in clinical samples from an endemic area of Brazil and highlight the influence of extraction methods, enzyme selection, and sample type on diagnostic accuracy. These observations reinforce the need for protocol standardization and multicenter validation studies before the widespread implementation of LAMP-based assays for the diagnosis of cutaneous leishmaniasis.

The lower specificity observed under some experimental conditions may be explained by a combination of technical and biological factors. First, the 18S rDNA target is a multicopy, highly conserved genomic region that contributes to the high analytical sensitivity of LAMP assays but may also increase the likelihood of non-specific amplification and cross-reactivity when reaction conditions are not fully optimized [22]. This interpretation is supported by our analytical specificity results, in which occasional cross-reactivity was observed with non-*Leishmania* pathogens. Similar limitations have been reported for molecular assays targeting highly conserved genomic regions, where increased sensitivity may come at the expense of reduced specificity.

In addition, subjective interpretation of colorimetric reactions may have contributed to discrepant results. Colorimetric LAMP assays rely on visual assessment of color changes, which can be challenging when reactions are weak or borderline, potentially leading to false-positive interpretations. This possibility is supported by analytical specificity experiments, in which discrepancies were observed between visual inspection and gel electrophoresis confirmation. Previous studies have highlighted the importance of objective result interpretation when colorimetric detection methods are employed. Although colorimetric LAMP assays are particularly attractive for point-of-care applications because they do not require specialized equipment, visual assessment may introduce observer-dependent variability. The future implementation of automated image-analysis systems, portable optical readers, or smartphone-based applications may improve result standardization, reduce interpretation bias, and enhance reproducibility, particularly in field settings and resource-limited environments [17,31].

Another factor that may have influenced assay performance is DNA quality. The success of molecular amplification methods depends on the integrity, purity, and concentration of extracted nucleic acids [27]. Differences among extraction protocols may affect DNA recovery and the removal of amplification inhibitors, thereby influencing diagnostic accuracy [32]. Consequently, variations in DNA quality obtained using different extraction approaches may partially explain the differences observed among the evaluated protocols. Together, these findings underscore the importance of target selection, optimization of reaction conditions, standardized DNA extraction procedures, and careful interpretation of colorimetric results to maximize the diagnostic performance of LAMP assays for cutaneous leishmaniasis.

Colorimetric detection using SYBR^®^ Green I proved to be a reliable and easily interpretable method, with clear visual differentiation between positive and negative results. In contrast, the WS colorimetric system produced intermediate color changes that were sometimes difficult to interpret, representing a limitation of the study and a potential barrier for point-of-care application. This challenge has also been described in previous studies using similar systems.

Finally, the limitations of this study include the number of confirmed cases, which was slightly below the target sample size. However, the study still provides valuable preliminary evidence regarding the performance of LAMP in swab samples. Other limitations are reduced specificity in some protocols, potential cross-reactivity associated with the 18S rDNA target, and variability in duplicate agreement, indicating issues with reproducibility. These findings highlight the need for further optimization of assay conditions, improvement of primer design, and enhanced standardization to maximize the diagnostic potential of LAMP for CL.

## 5. Conclusions

The present findings suggest that 18S rDNA-based LAMP has potential as a complementary diagnostic tool for cutaneous leishmaniasis. Among the evaluated protocols, the best performance was achieved using lesion swab samples, DNA extracted with the PureLink^®^ Genomic DNA Mini Kit, and amplification with Bst polymerase combined with SYBR Green detection. This protocol demonstrated the most favorable balance between sensitivity and operational feasibility. However, the moderate diagnostic performance observed under other experimental conditions, together with variability related to sample processing and result interpretation, highlights the need for further assay optimization. Future studies involving larger cohorts, standardized methodologies, and independent validation in different epidemiological settings will be essential to establish the robustness, reproducibility, and clinical applicability of this approach before its routine implementation in clinical practice. Notably, the best-performing protocol employed lesion swabs, a minimally invasive sampling approach that may facilitate implementation in field and point-of-care settings.

## Figures and Tables

**Table 1:** Clinical and demographic characteristics of participants of the study clinically suspected of cutaneous leishmaniasis, Rio de Janeiro, October 2021–December 2024.

Description	Profile	All Participants		Cases		Controls		*p*-Value
		N = 93	%	n = 42	%	n = 51	%	
Gender								
	Female	46	49.5	20	47.6	26	51.0	0.747
	Male	47	50.5	22	52.4	25	49.0	
Number of lesions								
	One	71	76.3	36	85.7	35	68.6	0.129
	Two or more	14	15.1	4	9.5	10	19.6	
	No information	8	8.6	2	4.8	6	11.8	
Lesion location								
	Lower limbs	38	40.9	20	47.6	18	35.3	0.077
	Upper limbs	23	24.7	14	33.3	9	17.6	
	Multiple locations	7	7.5	1	2.4	6	11.8	
	Other	16	17.2	5	11.9	11	21.6	
	No information	9	9.7	2	4.8	7	13.7	
		Median	Min–Max	Median	Min–Max	Median	Min–Max	
Age (years)		52	12.0–86.0	55	17–78	50	12.0–86	0.842
Evolution time (months) *		3	0.5–84.0	3	0.5–36	3	0.5–84	0.153

N = total number of participants included in the study; n = total number of participants per group (cases and controls); Min.: minimum value; Max.: maximum value; * twelve participants without this information.

**Table 2:** Median, interquartile range and minimum–maximum values of DNA quantification (ng/µL), measured by NanoDrop, and 260/280 purity ratio in clinical samples from patients with suspected cutaneous leishmaniasis, Rio de Janeiro, Brazil, from October 2021 to December 2024.

Clinical Sample	Median	IQR	Minimum–Maximum
Biopsy specimen			
Quantification	26.9	11–60	1–301.3
260/280	1.97	1.87–2.15	1.61–5.2
SW-kit			
Quantification	17.0	7.6–32.9	0.5–241.8
260/280	1.85	1.79–1.95	0.85–4.14
SW-DB			
Quantification	687	327.8–1261.3	65–1603
260/280	0.65	0.52–0.74	0.45–0.93

IQR, interquartile range; SW-kit, cutaneous lesion swab with DNA extracted using the PureLink^®^ Genomic DNA Mini Kit; SW-DB, cutaneous lesion swab with DNA extracted using the boiling method.

**Table 3:** Diagnostic performance of the diagnostic tests LAMP 18S rDNA assays using WarmStart^®^ Colorimetric LAMP 2× Master Mix or Bst 2.0 enzyme with SYBR Green, according to clinical samples type.

Clinical Sample	Sensitivity % (95% CI)	Specificity % (95% CI)	Accuracy % (95% CI)
LAMP 18S rDNA (WS)			
SW-kit (n = 84)	52.5 (36.1–68.5)	86.4 (72.6–94.8)	70.2 (59.3–79.7)
SW-DB (n = 85)	67.6 (50.2–82.0)	39.6 (25.8–54.7)	51.8 (40.7–62.7)
Biopsy (n = 93)	59.5 (43.3–74.4)	74.0 (59.7–85.4)	67.4 (56.8–76.8)
LAMP 18S rDNA (BstSg)			
SW-kit (n = 84)	80.0 (64.4–90.9)	77.3 (62.2–88.5)	78.6 (68.3–86.8)
SW-DB (n = 85)	54.1 (36.9–70.5)	77.1 (62.7–88.0)	67.1 (56.0–76.9)
Biopsy (n = 93)	81.0 (65.9–91.4)	68.0 (53.3–80.5)	73.9 (63.7–82.5)

WS, WarmStart^®^ Colorimetric LAMP 2× Master Mix; BstSg, Bst 2.0 enzyme with SYBR Green intercalating dye; SW-kit: DNA extracted from cutaneous lesion swabs using the PureLink^®^ Genomic DNA Mini Kit; SW-DB: DNA extracted from cutaneous lesion swabs by the direct boiling method; Biopsy: biopsy specimen; CI, confidence interval; n, number of clinical samples analyzed.

## Data Availability

The raw data supporting the conclusions of this article will be made available by the authors on request.

## Supplementary Materials

The following supporting information can be downloaded at https://www.mdpi.com/article/10.3390/tropicalmed11070207/s1. Table S1. Analytical specificity of the 18S rDNA LAMP assays using WarmStart^®^ Colorimetric LAMP 2× Master Mix (WS) and Bst 2.0 DNA Polymerase with SYBR^®^ Green (BstSg), evaluated by visual inspection and agarose gel electrophoresis.

## References

[1] World Health Organization Leishmaniasis. https://www.who.int/news-room/fact-sheets/detail/Leishmaniasis.

[2] Gabriel Á., Valério-Bolas A., Palma-Marques J., Mourata-Gonçalves P., Ruas P., Dias-Guerreiro T., Santos-Gomes G. (2019). Cutaneous Leishmaniasis: The Complexity of Host’s Effective Immune Response against a Polymorphic Parasitic Disease. J. Immunol. Res..

[3] Gurel M.S., Tekin B., Uzun S. (2020). Cutaneous Leishmaniasis: A great imitator. Clin. Dermatol..

[4] Goto H., Lindoso J.A.L. (2010). Current diagnosis and treatment of cutaneous and mucocutaneous Leishmaniasis. Expert Rev. Anti-Infect. Ther..

[5] Jarzabek J., Denny P.W. (2025). Molecular diagnostics for cutaneous Leishmaniasis: Progress towards fulfilling the WHO target product profile. Parasitology.

[6] Nzelu C.O., Gomez E.A., Cáceres A.G., Sakurai T., Martini-Robles L., Uezato H., Mimori T., Katakura K., Hashiguchi Y., Kato H. (2014). Development of a loop-mediated isothermal amplification method for rapid mass-screening of sand flies for *Leishmania* infection. Acta Trop..

[7] Banoo S., Bell D., Bossuyt P., Herring A., Mabey D., Poole F., Smith P.G., Sriram N., Wongsrichanalai C., Linke R. (2008). Evaluation of diagnostic tests for infectious diseases: General principles. Nat. Rev. Microbiol..

[8] Faria V.C.S., Gonçalves D.U., Soares A.R.C., Barbosa P.H., Saliba J.W., Souza C.S.A., Cota G.F., Avelar D.M. (2022). Impact assessment of different DNA extraction methods for non-invasive molecular diagnosis of tegumentary Leishmaniasis. Acta Trop..

[9] Fabiano-Coelho L., Santos F.N., Miranda L.D.F.C. (2025). Standardization of the Miniculture Technique for Isolation of Leishmania spp..

[10] Cupolillo E., Grimaldi G., Momen H. (1994). A general classification of New World *Leishmania* using numerical zymotaxonomy. Am. J. Trop. Med. Hyg..

[11] de Mello C.X., Madeira M.d.F. (2015). Skin scraping is the most accessible technique for the parasitological diagnosis of American tegumentary Leishmaniasis. Am. J. Trop. Med. Hyg..

[12] Quintella L.P., Cuzzi T., Madeira M.D.F., Okamoto T., Schubach A.D.O. (2009). Immunoperoxidase technique using an anti-*Leishmania* (L.) chagasi hyperimmune serum in the diagnosis of culture-confirmed American tegumentary Leishmaniasis. Rev. Inst. Med. Trop. São Paulo.

[13] Fagundes A., Schubach A., de Paula C.C., Bogio A., Antonio L.d.F., Schiavoni P.B., Monteiro V.d.S., Madeira M.d.F., Quintella L.P., Valete-Rosalino C.M. (2010). Evaluation of polymerase chain reaction in the routine diagnosis for tegumentary Leishmaniasis in a referral centre. Mem. Inst. Oswaldo Cruz.

[14] Mikita K., Maeda T., Yoshikawa S., Ono T., Miyahira Y., Kawana A. (2014). The Direct Boil-LAMP method: A simple and rapid diagnostic method for cutaneous Leishmaniasis. Parasitol. Int..

[15] Saiki R.K., Gelfand D.H., Stoffel S., Scharf S.J., Higuchi R., Horn G.T., Mullis K.B., Erlich H.A. (1988). Primer-directed enzymatic amplification of DNA with a thermostable DNA polymerase. Science.

[16] Adams E.R., Schoone G.J., Ageed A.F., El Safi S., Schallig H.D.F.H. (2010). Development of a Reverse Transcriptase Loop-Mediated Isothermal Amplification (LAMP) Assay for the Sensitive Detection of *Leishmania* Parasites in Clinical Samples. Am. J. Trop. Med. Hyg..

[17] Nzelu C., Kato H., Peters N. (2019). Loop-mediated isothermal amplification (LAMP): An advanced molecular point-of-care technique for the detection of *Leishmania* infection. PLoS Neglected Trop. Dis..

[18] León C.M., Muñoz M., Tabares J.H., Hernandez C., Florez C., Ayala M.S., Ramírez J.D. (2018). Analytical Performance of a Loop-Mediated Isothermal Amplification Assay for *Leishmania* DNA Detection in Sandflies and Direct Smears of Patients with Cutaneous Leishmaniasis. Am. J. Trop. Med. Hyg..

[19] Filgueira C.P.B., Moreira O.C., Cantanhêde L.M., De Farias H.M.T., Porrozzi R., Britto C., Boité M.C., Cupolillo E. (2020). Comparison and clinical validation of QPCR assays targeting *Leishmania* 18s rDNA and HSP70 genes in patients with american tegumentary Leishmaniasis. PLoS Neglected Trop. Dis..

[20] Moreira O.C., Yadon Z.E., Cupolillo E. (2018). The applicability of real-time PCR in the diagnostic of cutaneous Leishmaniasis and parasite quantification for clinical management: Current status and perspectives. Acta Trop..

[21] Notomi T., Okayama H., Masubuchi H., Yonekawa T., Watanabe K., Amino N., Hase T. (2000). Loop-mediated isothermal amplification of DNA. Nucleic Acids Res..

[22] Adams E.R., Schoone G., Versteeg I., Gomez M.A., Diro E., Mori Y., Perlee D., Downing T., Saravia N., Assaye A. (2018). Development and Evaluation of a Novel Loop-Mediated Isothermal Amplification Assay for Diagnosis of Cutaneous and Visceral Leishmaniasis. J. Clin. Microbiol..

[23] Celeste JLde L., Caldeira R.L., Pires Sda F., Silveira K.D., Soares R.P., de Andrade H.M. (2019). Development and evaluation of a loop-mediated isothermal amplification assay for rapid detection of *Leishmania* amazonensis in skin samples. Exp. Parasitol..

[24] Salari S., Taghdiri A., Bamorovat M., Sharifi I., Ghasemi Nejad Almani P. (2022). A novel rapid LAMP test for identification of cutaneous Leishmaniasis: An evaluation and comparative analysis of three molecular methods. Microb. Pathog..

[25] Soares A.R.C., de Faria V.C.S., de Avelar D.M. (2024). Development and accuracy evaluation of a new loop-mediated isothermal amplification assay targeting the HSP70 gene for the diagnosis of cutaneous Leishmaniasis. PLoS ONE.

[26] Blaizot R., Simon S., Ginouves M., Prévot G., Blanchet D., Ravel C., Couppie P., Demar M., Nabet C. (2021). Validation of swab sampling and SYBR green-based real-time PCR for the diagnosis of cutaneous Leishmaniasis in French Guiana. J. Clin. Microbiol..

[27] Lucena-Aguilar G., Sánchez-López A.M., Barberán-Aceituno C., Carrillo-Ávila J.A., López-Guerrero J.A., Aguilar-Quesada R. (2016). DNA Source Selection for Downstream Applications Based on DNA Quality Indicators Analysis. Biopreserv. Biobank..

[28] Aronson N.E., Joya C.A. (2019). Cutaneous Leishmaniasis: Updates in Diagnosis and Management. Infect. Dis. Clin..

[29] Erber A.C., Sandler P.J., de Avelar D.M., Swoboda I., Cota G., Walochnik J. (2022). Diagnosis of visceral and cutaneous Leishmaniasis using loop-mediated isothermal amplification (LAMP) protocols: A systematic review and meta-analysis. Parasites Vectors.

[30] Weirather J.L., Jeronimo S.M., Gautam S., Sundar S., Kang M., Kurtz M.A., Haque R., Schriefer A., Talhari S., Carvalho E.M. (2011). Serial quantitative PCR assay for detection, species discrimination, and quantification of *Leishmania* spp. in human samples. J. Clin. Microbiol..

[31] Priye A., Bird S.W., Light Y.K., Ball C.S., Negrete O.A., Meagher R.J. (2017). A smartphone-based diagnostic platform for rapid detection of Zika, chikungunya, and dengue viruses. Sci. Rep..

[32] Schrader C., Schielke A., Ellerbroek L., Johne R. (2012). PCR inhibitors—Occurrence, properties and removal. J. Appl. Microbiol..

