# Supplementary material for: Evaluation of the Loop-Mediated Isothermal Amplification (LAMP) Technique in Swab Samples from Ulcerated Cutaneous Lesions Compared with Conventional Diagnostic Methods for American Tegumentary Leishmaniasis in Patients Treated at a Reference Center in Rio de Janeiro, Brazil
Source: Trop Med Infect Dis. 2026 Jul 22;11(7):207. doi: 10.3390/tropicalmed11070207 (PMC13417201; doi:10.3390/tropicalmed11070207)
Supplement: Supplementary file 1 [file tropicalmed-11-00207-s001.zip › tropicalmed-4443259-supplementary.pdf]

Supplementary Table S1. Analytical specificity of the 18S rDNA LAMP assays using WarmStart® Colorimetric LAMP 2× Master Mix (WS) and Bst 2.0 DNA Polymerase with SYBR® Green (BstSg), evaluated by visual inspection and agarose gel electrophoresis.

Organism	WS (visual)	WS (gel)	BstSg (visual)	BstSg (gel)
L. braziliensis	+	+	+	+
L. amazonensis	+	+	-	+
L. infantum	+	+	-	+
L. guyanensis	+	+	-	+
Paracoccidioides brasiliensis	+ (1/2)	+ (1/2)	-	-
Sporothrix globosa	-	+ (1/2)	-	-

WS: WarmStart® Colorimetric LAMP 2× Master Mix; BstSg: Bst 2.0 DNA Polymerase with SYBR® Green I dye; Pb: *Paracoccidioides brasiliensis*; Sg: *Sporothrix globosa*; Lb: *Leishmania (Viannia) braziliensis*; Li: *Leishmania (Leishmania) infantum*; Lg: *Leishmania (Viannia) guyanensis*; La: *Leishmania (Leishmania) amazonensis*. The results are presented according to the visual inspection of color change and confirmation by agarose gel electrophoresis. Positive reactions that were only observed in one of the duplicate assays are indicated as (+/−).
